# Esculin and esculetin protect against bone loss in osteoporosis by rebalancing osteoblast and osteoclast functions

**DOI:** 10.3389/fphar.2026.1961311

**Published:** 2026-10-26

**Authors:** Lei Tong, Weizhen Lv, Huaqing Liu

**Affiliations:** 1 Department of Orthopedic Trauma, Nanyang Central Hospital, Nanyang, Henan, China; 2 Department of Gastroenterology and General Surgery, Nanyang Central Hospital, Nanyang, Henan, China

**Keywords:** bone remodeling, combination therapy, esculetin, esculin, osteoporosis

## Abstract

**Background:**

Osteoporosis is characterized by uncoupled bone remodeling where resorption outpaces formation. This study investigated the roles of two natural coumarins, Esculin (ECL) and Esculetin (ECLT), in bone metabolism.

**Methods:**

We evaluated the bidirectional effects of ECL and ECLT on primary osteoblasts and RAW264.7-derived osteoclasts *in vitro*. *In vivo*, the translational protective efficacy of the compounds was assessed using ovariectomized (OVX) and sciatic nerve transection (DEN) mouse models, representing postmenopausal and disuse osteoporosis, respectively. Evaluations included serum biochemical analysis, histological staining, micro-computed tomography (Micro-CT), and three-point bending biomechanical tests.

**Results:**

*In vitro* assays identified optimal pharmacological concentrations of 50 μM for ECL and 10 μM for ECLT, with both compounds dose-dependently promoting osteoblast proliferation and mineralization while simultaneously inducing osteoclast apoptosis and downregulating key resorptive genes. Co-administration of ECL and ECLT demonstrated enhanced efficacy, greater than either monotherapy in several key readouts. In both OVX and DEN *in vivo* models, the combinatorial regimen markedly ameliorated systemic inflammation and oxidative stress, and successfully rebalanced circulating bone turnover markers. Furthermore, the dual therapy robustly prevented the deterioration of three-dimensional trabecular and cortical bone microarchitecture and substantially improved the functional biomechanical strength of the femurs.

**Conclusion:**

The concurrent application of ECL and ECLT protects against bone loss by concurrently promoting osteoblast function and suppressing osteoclast formation, representing a promising bidirectional strategy for osteoporosis management.

## Introduction

Osteoporosis is a prevalent systemic skeletal disorder characterized by the progressive deterioration of bone microarchitecture, reduced bone mineral density (BMD), and a concomitantly increased risk of fragility fractures (Liu C. et al., 2026; Wang F. et al., 2026). The fundamental pathogenesis of osteoporosis revolves around the disruption of bone homeostasis, a delicate and dynamic remodeling process orchestrated by bone-forming osteoblasts and bone-resorbing osteoclasts (Jin et al., 2026; Li et al., 2026). Under normal physiological conditions, bone resorption and formation are tightly coupled (Zhou et al., 2026). However, a variety of pathological triggers, including estrogen deficiency, aging, lack of mechanical stimulation, and oxidative stress, can disrupt this equilibrium (Liu et al., 2025; Cai et al., 2026; Liu M. et al., 2026). Despite their distinct initial triggers, these diverse etiologies ultimately converge on a common pathophysiological pathway: the overactivation of osteoclast-mediated bone resorption that drastically outpaces osteoblast-mediated bone formation (An et al., 2026; Xi et al., 2026). This uncoupling results in continuous net bone loss, underscoring the necessity of targeting the fundamental cellular machineries of both osteoclasts and osteoblasts to effectively halt disease progression.

Currently, the pharmacological management of osteoporosis primarily relies on antiresorptive agents, such as bisphosphonates and denosumab, or anabolic agents like teriparatide (Pascual-Pastor and Barceló-Bru, 2026; Salman et al., 2026; Sheikh, 2026). While these therapies are highly effective in increasing BMD, their long-term clinical application is often limited by severe adverse effects, including osteonecrosis of the jaw, atypical femoral fractures, and gastrointestinal toxicity (Sheikh, 2026; Tskhakaia and Danila, 2026; Uddin et al., 2026; Wang M. et al., 2026). Furthermore, most existing drugs target only one aspect of bone remodeling, often leading to a secondary suppression of the opposing process (Wang Y. et al., 2026; Wang F. et al., 2026). Consequently, there is an urgent and unmet clinical need to identify novel, natural compound-derived therapeutics that can safely and simultaneously promote osteogenesis and inhibit osteoclastogenesis.

Esculin (ECL) and its aglycone derivative, Esculetin (ECLT), are naturally occurring coumarin compounds primarily isolated from Cortex Fraxini (Qinpi), a traditional botanical medicine widely utilized for its anti-inflammatory, antioxidant, and immunomodulatory properties (Li et al., 2022; Cai and Cai, 2023). Recent pharmacological investigations have increasingly highlighted the protective effects of coumarin derivatives in metabolic and degenerative diseases (Guo et al., 2025; Chen et al., 2026; Gamil et al., 2026). However, despite preliminary evidence suggesting that natural coumarins may influence cellular microenvironments, the specific pharmacological roles of ECL and ECLT in the intricate regulatory networks of bone metabolism remain incompletely understood. Specifically, a systematic comparison of their therapeutic efficacies, optimal pharmacological concentrations, and dual actions on osteoblast and osteoclast lineages has yet to be elucidated. Furthermore, to rigorously validate the translational potential of these compounds, their osteoprotective efficacy must be evaluated across different experimental models that simulate various clinical etiologies of bone loss. Addressing this gap is critical for establishing a comprehensive pharmacological profile of ECL and ECLT.

Therefore, the present study was designed to systematically investigate the pharmacological effects and underlying mechanisms of ECL and ECLT on bone metabolism. *In vitro*, we evaluated their dual regulatory actions on osteoclastogenesis and osteoblastogenesis, meticulously screening and confirming the optimal therapeutic concentration windows for both compounds. *In vivo*, we employed both ovariectomized (OVX) and sciatic nerve transection (DEN) models to comprehensively and robustly evaluate the protective efficacy of ECL and ECLT against distinct osteoporotic conditions. Ultimately, this study aims to provide solid preclinical evidence supporting the therapeutic potential of ECL and ECLT as novel, bidirectional agents for the prevention and treatment of osteoporosis.

## Methods

### Cell culture and reagents

Primary mouse calvarial osteoblasts were isolated from neonatal C57BL/6J mice (n = 10–15 pooled pups per isolation, 1–3 days old, both sexes due to the difficulty of neonatal sexing) using sequential collagenase digestion. Cells from passages two to 3 (P2–P3) were used for all subsequent experiments to maintain primary characteristics. Osteoclasts were differentiated from the RAW264.7 murine macrophage cell line upon stimulation with 50 ng/mL receptor activator of nuclear factor-κB ligand (RANKL) for 4–6 days. Mature osteoclast formation was confirmed by the presence of large, multinucleated (≥3 nuclei) tartrate-resistant acid phosphatase (TRAP)-positive cells. Cells were cultured in a standard growth medium supplemented with 10% fetal bovine serum (FBS) and 1% penicillin/streptomycin in a humidified incubator at 37 °C with 5% CO_2_. Esculin (ECL) and Esculetin (ECLT) of high purity (≥98%) were dissolved in dimethyl sulfoxide (DMSO) and diluted in phosphate-buffered saline (PBS) for *in vitro* experiments. The final concentration of DMSO in the culture media never exceeded 0.1% (v/v) to strictly preclude vehicle-induced cytotoxicity.

### *In Vitro* experimental design and concentration optimization

To evaluate the dose-dependent effects of the compounds and determine the optimal pharmacological concentration, osteoblasts and RAW264.7-derived osteoclasts were treated with varying concentrations of ECL and ECLT (0, 1, 5, 10, 50, and 100 μM). Following the identification of the optimal concentration via viability and functional assays, subsequent *in vitro* experiments were divided into four distinct groups: the PBS vehicle control group, the ECL monotherapy group, the ECLT monotherapy group, and the combined ECL + ECLT treatment group.

### Cell viability and proliferation assays

Osteoblast viability was assessed using the MTT assay. Briefly, osteoblasts were seeded in 96-well plates at a density of 5 × 10^3^ cells/well and treated with the indicated concentrations of ECL and ECLT for 48 h. After the respective incubation periods, MTT solution was added, and the generated formazan crystals were dissolved in DMSO. Absorbance was measured using a microplate reader at 490 nm. To evaluate osteoblast proliferation, an EdU incorporation assay was performed using flow cytometry. Cells were seeded in 6-well plates at 1 × 10^5^ cells/well and treated for 48 h. Cells were incubated with EdU solution, harvested, fixed, permeabilized, and subjected to the Click-iT reaction according to the manufacturer’s instructions. During flow cytometry analysis, debris and doublets were excluded via standard forward scatter (FSC) and side scatter (SSC) gating strategies. The percentage of EdU-positive proliferating osteoblasts was then quantified using a flow cytometer, and data were analyzed with FlowJo software.

### Osteoclast apoptosis analysis

Osteoclast apoptosis was quantified using an Annexin V/Propidium Iodide (PI) double-staining approach via flow cytometry. Briefly, RAW264.7-derived osteoclasts (seeded at 2 × 10^5^ cells/well in 6-well plates) treated with ECL and/or ECLT for 24 h were harvested and washed with cold PBS. Cells were then stained with Annexin V using an Annexin V-FITC/PI apoptosis detection kit according to the manufacturer’s protocol. Single-color stained cells were used to establish compensation settings. Stained cells were subsequently analyzed using a BD FACS flow cytometer to determine the proportions of early (Annexin V+/PI-) and late (Annexin V+/PI+) apoptotic cells.

### Osteogenic differentiation and Alizarin Red S (ARS) staining

To evaluate the mineralization capacity, osteoblasts were seeded in complete osteogenic induction medium (supplemented with 50 μg/mL ascorbic acid, 10 mM β-glycerophosphate, and 100 nM dexamethasone). Following treatment with the optimized concentrations of ECL, ECLT, or their combination for exactly 21 days, the cells were fixed in 4% paraformaldehyde (PFA) and stained with 2% Alizarin Red S (ARS) solution (pH 4.2) for 15 min at room temperature. The mineralized nodules were photographed. For quantitative analysis, the percentage of the ARS-positive stained area relative to the total well area was measured using ImageJ software (NIH).

#### RNA isolation and real-time quantitative PCR (RT-qPCR)

Total RNA was extracted from cultured cells using an RNAeasy Animal RNA Isolation Kit with a spin column according to the manufacturer’s instructions. Complementary DNA (cDNA) was synthesized using PrimeScript RT Master Mix. Subsequently, gene expression was measured by RT-qPCR analysis using BeyoFast SYBR Green qPCR Mix and the LightCycler 480 II. The experimental conditions involved 40 cycles of denaturation at 95 °C for 15 s and amplification at 60 °C for 1 min. Relative mRNA expression levels of target genes, including osteogenic and osteoclastogenic markers, were analyzed using the 2^-ΔΔCt^ method, with data normalized to Gapdh expression. The primer sequences used in this study are detailed in Supplementary Table S1. All primers were validated to ensure amplification efficiencies between 90% and 110% prior to final quantification.

### Animal models and experimental grouping

All animal experiments were approved by the Institutional Animal Care and Use Committee (Animal Research Reviewers No. 2025M1SH, approved in January 2025) and performed in strict accordance with ethical guidelines. Eight-week-old female C57BL/6J mice (average body weight: 20–22 g) were housed in a specific pathogen-free facility at 23 °C ± 2 °C with a 12-h light/dark cycle and free access to standard chow and water. To simulate postmenopausal osteoporosis, mice underwent bilateral ovariectomy (OVX) under anesthesia. To simulate disuse osteoporosis, a denervation (DEN) model was established where the right sciatic nerve was exposed, and a 5-mm segment was excised to induce denervation. Successful ovariectomy was verified at necropsy by the absence of ovarian tissue and marked uterine atrophy, and successful denervation was reflected by visible atrophy of the denervated hindlimb musculature. Sham-operated mice served as controls for both models. Anesthesia was induced with 4% isoflurane and maintained with 1.5%–2.5% isoflurane; surgical-plane anesthesia was confirmed by the absence of a pedal withdrawal reflex. Postoperative analgesia (subcutaneous buprenorphine, 0.1 mg/kg) was administered for 3 days to alleviate pain. Animals were closely monitored post-surgery, and humane endpoints were established based on severe weight loss or signs of overt distress. Two weeks following successful surgical modeling, the mice were randomly allocated into their respective groups (Vehicle, ECL, ECLT, and ECL + ECLT) using a computer-generated randomization sequence with concealed allocation. The sample size for each group was determined based on comparable previous studies in bone research, with the specific number of animals (n) detailed in the respective figure legends. No animals were excluded during the experiment, and no unexpected mortality or adverse events occurred; thus, all initial animals were included in the downstream analyses. Specifically, ECL was administered at a dose of 40 mg/kg once daily (Sun et al., 2024), while ECLT was given at 10 mg/kg once every 3 days (Kim et al., 2026). The combined treatment group received both compounds at their respective doses and frequencies, and the Vehicle control group received an equal volume of the solvent. The compounds were uniformly suspended in a vehicle composed of 0.5% sodium carboxymethyl cellulose, and all treatments, including the Vehicle control, were administered at a standardized volume of 10 mL/kg body weight. All treatments were maintained for a continuous duration of 8 weeks. On the day following the final administration, all mice were euthanized, and the relevant tissues were harvested for subsequent downstream analyses. For euthanasia, mice were first deeply anesthetized with 5% isoflurane; then, while still under deep anesthesia, they were killed by cervical dislocation. The right femurs were harvested for all bone-related assessments. To prevent tissue processing artifacts, mice per group were divided into two subsets. One subset was used for fresh bone biomechanical testing, while the other was fixed in 4% PFA for Micro-CT and subsequent histological analysis. For ELISA, serum was randomly collected from eight mice per group prior to euthanasia. Notably, treatment administration, histological evaluations, micro-CT scanning, and statistical analyses were conducted by independent investigators strictly blinded to the experimental group allocations.

### Enzyme-Linked Immunosorbent Assay (ELISA)

Serum samples were collected to evaluate the systemic levels of bone turnover markers, inflammatory cytokines, and oxidative stress indicators. The concentrations of these markers were quantified using specific Enzyme-Linked Immunosorbent Assay (ELISA) kits according to the manufacturer’s instructions. Specifically, the following functional panels were evaluated: bone formation markers, including procollagen I C-terminal propeptide (PICP, #JL10121), procollagen I N-terminal propeptide (PINP, #JL20174), and bone alkaline phosphatase (BALP, #JL20367); bone resorption markers, including N-terminal telopeptide of type I collagen (NTX-I, #JL15569), C-terminal telopeptide of type I collagen (CTX-I, #JL20123), and tartrate-resistant acid phosphatase (TRAP, #JL20424); inflammatory cytokines, including tumor necrosis factor-alpha (TNF-α, #JL10484), interleukin-1 beta (IL-1β, #JL18442), interleukin-6 (IL-6, #JL20268), and interleukin-10 (IL-10, #JL20242); and oxidative stress indicators, including superoxide dismutase (SOD, #JL12237) and malondialdehyde (MDA, #JL53632). All aforementioned ELISA kits were purchased from JonlnBio (Shanghai, China). Following the chromogenic reactions, the absorbance was measured using a microplate reader set at 450 nm with a wavelength correction of 540 nm.

### Histological and immunofluorescence analysis

For histological evaluation, femurs were fixed in 4% PFA for 48 h, decalcified in 17% ethylenediaminetetraacetic acid (EDTA) for 21 days at room temperature with regular changes of solution, and then embedded in paraffin. Serial sections (5-μm thick) were cut along the sagittal plane of the distal femur. For anatomical consistency, sections were sampled starting from the central weight-bearing region. Routine Hematoxylin and Eosin (H&E), Masson’s trichrome, and Toluidine blue staining were performed to evaluate general bone architecture, collagen deposition, and cartilage/bone transitional zones, respectively. Tartrate-resistant acid phosphatase (TRAP) staining was performed using a commercial kit according to the manufacturer’s instructions to identify osteoclasts. For immunofluorescence (IMF), decalcified femur sections were deparaffinized and subjected to antigen retrieval in citrate buffer for 20 min. Sections were blocked with 10% normal goat serum for 1 h at room temperature, then incubated overnight with primary antibodies against Collagen Type I (COL1, #ab316222; Abcam) and Osteocalcin (OCN, #23418-1-AP; Proteintech). After washing, sections were incubated with Alexa Fluor 594-conjugated secondary antibodies for 1 h at room temperature, and nuclei were counterstained with DAPI. Images were acquired using a confocal laser scanning microscope. All microscopic images were captured at standard magnifications (×40 or 80×, with corresponding scale bars of 100 μm or 50 μm, respectively). For quantitative histomorphometry, three representative non-consecutive sections per animal were selected, and three random fields within the secondary spongiosa (1–2 mm proximal to the epiphyseal growth plate) were analyzed per section using ImageJ software (NIH, Bethesda, MD, USA). All imaging and quantifications were performed by investigators blinded to the treatment groups.

#### Micro-Computed Tomography (Micro-CT) analysis

The distal femurs were scanned using a high-resolution micro-CT imaging device (Quantum GX, PerkinElmer) at a voxel size of 10 μm, voltage of 90 kV, and current of 88 μA. After scanning, 3D image reconstruction was performed using NRecon software with standard beam-hardening correction and ring artifact reduction applied. Quantitative analysis was conducted via CTAn software. The region of interest (ROI) for trabecular bone was defined as commencing 0.5 mm proximal to the distal growth plate of the femur and extending exactly 2.0 mm in a proximal direction, encompassing the secondary spongiosa. For cortical bone, an ROI of 1.0 mm in length was selected at the femoral mid-diaphysis. Bone tissue was segmented from marrow and soft tissue using a global thresholding algorithm with a fixed numerical threshold of 85 (gray values), following calibration with standard hydroxyapatite phantoms. The following trabecular and cortical bone parameters were quantified in accordance with the American Society for Bone and Mineral Research (ASBMR) guidelines: bone volume fraction (BV/TV, %), trabecular thickness (Tb.Th, mm), trabecular separation (Tb.Sp, mm), trabecular number (Tb.N, 1/mm), cortical bone thickness (Cb.Th, mm), and connectivity density (Conn.D, mm^-3^).

#### Biomechanical analysis

Prior to testing, harvested femurs were wrapped in saline-soaked gauze and stored at −20 °C. On the day of testing, samples were thawed at room temperature and kept continuously hydrated in physiological saline. The femurs underwent a three-point bending test using an Instron 3366 Universal Tabletop Testing System to evaluate biomechanical properties. The femur was positioned on a support device in an anterior-posterior orientation with two loading points 8.0 mm apart to preserve its natural curvature. Mechanical loading was applied to the anterior mid-shaft of the diaphysis at a constant displacement rate of 2 mm/min until fracture. Before testing, the anterior-posterior and mediolateral diameters of the mid-diaphysis were measured using a digital caliper. The following biomechanical parameters were determined from the recorded load-displacement curves: maximum force (N), stiffness (N/mm), ultimate stress (MPa), and elastic modulus (MPa). Ultimate stress (σ) and elastic modulus (E) were calculated using standard engineering equations derived from Euler-Bernoulli beam theory: σ = (F × L × c)/(4 × I) and E = (S × L^3^)/(48 × I), where F is the maximum force, L is the span length (8.0 mm), c is the distance from the center of mass to the outer surface (half of the anterior-posterior diameter), S is the stiffness (slope of the linear elastic region), and I is the cross-sectional moment of inertia, calculated by modeling the mid-diaphysis as a hollow ellipse to account for the medullary cavity, using both the outer and inner anterior-posterior and mediolateral diameters.

#### Statistical analysis

R (version 4.5.2) was used for statistical analysis. Continuous variables were expressed as mean ± standard deviation (SD). Normality was assessed using the Shapiro–Wilk test and homogeneity of variances using Levene’s test; one-way analysis of variance (ANOVA) followed by Tukey’s *post hoc* multiple-comparison test was used for comparisons among more than two groups, as this approach is robust to the modest deviations from normality observed at these sample sizes. For the combination experiments, planned pairwise comparisons between the ECL + ECLT combination and each monotherapy were extracted from the same Tukey analysis and are summarized in Supplementary Table S2. Because a formal factorial interaction model was not pre-specified, the effects of the combination are consistently described as combined or enhanced rather than synergistic. For the variables that showed unequal variances according to Levene’s test, sensitivity analyses were additionally conducted using Welch’s ANOVA with Games–Howell *post hoc* comparisons, which do not assume equal variances. For outcomes showing substantial departures from normality, Kruskal–Wallis tests were further performed, followed by Dunn’s *post hoc* comparisons with Holm correction. The overall conclusions regarding the presence or absence of between-group differences were identical across the conventional ANOVA, Welch’s ANOVA, and Kruskal–Wallis approaches for all affected outcomes (Supplementary Table S3), confirming that the reported results are robust to violations of the homogeneity-of-variance and normality assumptions. Exact adjusted P values for all pairwise comparisons, together with mean differences and effect sizes (Cohen’s d), are provided in Supplementary Table S2. In all figures, asterisks denote comparisons with the respective control group (0 μM, PBS, OVX, or DEN): *P < 0.05, **P < 0.01, ***P < 0.001, ****P < 0.0001; ns, not significant. A p-value less than 0.05 was considered statistically significant. All *in vitro* experiments were repeated at least three times independently using distinct cell isolations. For all cell-based assays, each independent biological replicate (culture well) was subjected to three technical replicates. The values from these technical replicates were averaged to generate a single data point prior to statistical analysis. Technical replicates were not treated as independent observations. The sample size was summarized in the respective figure legends.

## Results

### Roles of ECL in osteoblasts and osteoclasts *in vitro*

To systematically evaluate the pharmacological effects of Esculin (ECL) on bone remodeling *in vitro*, we first investigated its impact on osteogenic differentiation across a gradient of concentrations (0, 1, 5, 10, 50, and 100 μM). Real-time qPCR analysis demonstrated that ECL treatment significantly upregulated the mRNA expression levels of key osteogenic marker genes in primary osteoblasts, including Alp, Col1a, Ocn, and Runx2 (Figure 1A). This transcriptional upregulation exhibited a dose-dependent pattern, reaching a plateau at the concentration of 50 μM. This pro-osteogenic effect was further visually and quantitatively corroborated by Alizarin Red S (ARS) staining, which showed that ECL enhanced extracellular matrix mineralization and calcium nodule formation, with the most robust mineral deposition likewise observed at 50 μM (Figure 1B). Subsequently, we assessed the effect of ECL on osteoblast proliferation and viability. The EdU incorporation assay, as illustrated by flow cytometry histograms (Figure 1C) and corresponding quantitative analysis (Figure 1D), revealed that ECL dose-dependently stimulated osteoblast proliferation, achieving a maximal stimulatory effect at 50 μM before exhibiting a slight decline at 100 μM. Consistently, the cell viability assay indicated that ECL exhibited no noticeable cytotoxicity to osteoblasts within the tested concentration range; instead, it maintained or slightly enhanced overall cell viability, peaking at 50 μM (Figure 1E). Functionally, this coordinated increase in osteogenic transcription, matrix mineralization, and proliferation indicates that ECL supports the anabolic arm of bone remodeling rather than a single surrogate marker, providing a cellular basis for the bone-forming effects observed *in vivo*.

**FIGURE 1:**
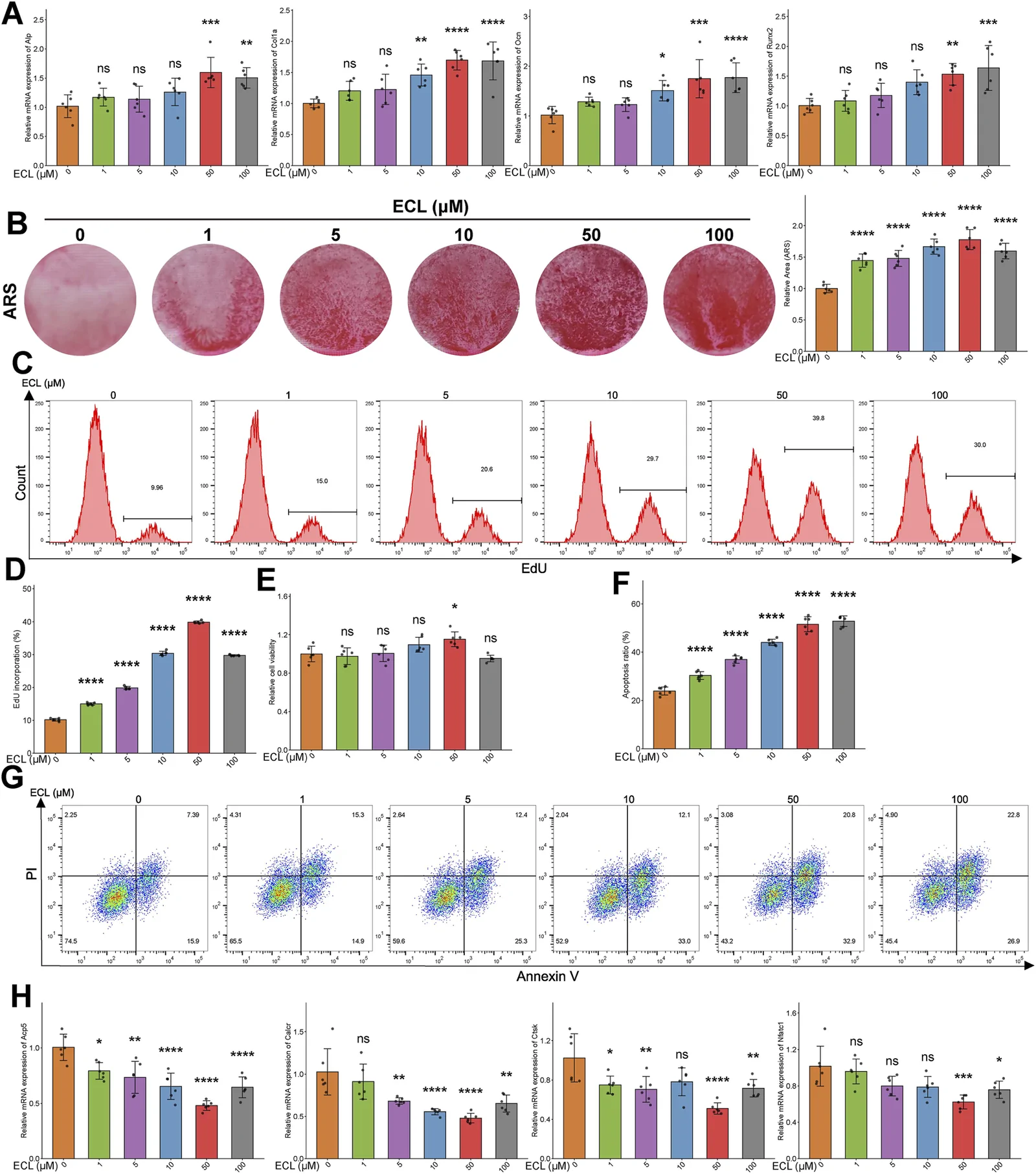
Esculin (ECL) dose-dependently promotes osteoblastogenesis and inhibits osteoclastogenesis *in vitro*. **(A)** Relative mRNA expression of osteogenic marker genes (Alp, Col1a, Ocn, and Runx2) in primary osteoblasts treated with varying concentrations of ECL. **(B)** Representative images and quantitative analysis of Alizarin Red S (ARS) staining. **(C,D)** Flow cytometry histograms **(C)** and quantification **(D)** of EdU incorporation reflecting osteoblast proliferation. **(E)** Osteoblast viability assessed by the MTT assay. **(F–G)** Quantitative apoptosis ratio **(F)** and representative flow cytometry dot plots **(G)** of RAW264.7-derived osteoclasts using Annexin V/PI double staining. **(H)** Relative mRNA expression of osteoclastogenic marker genes (Acp5, Calcr, Ctsk, and Nfatc1). n = 6 per group. Each dot represents an independent biological replicate (well), which represents the average value of three technical replicates, and data are presented as mean ± SD (error bars). Differences among groups were assessed by one-way ANOVA followed by Tukey’s *post hoc* test; asterisks denote comparisons with the vehicle control (0 μM): *P < 0.05, **P < 0.01, ***P < 0.001, ****P < 0.0001; ns, not significant. Exact adjusted P values are provided in Supplementary Table S2.

Parallelly, we explored the direct anti-catabolic effects of ECL on RAW264.7-derived osteoclasts. Flow cytometric analysis utilizing Annexin V/PI double staining revealed that ECL administration robustly induced osteoclast apoptosis. The flow cytometry dot plots and the corresponding quantitative apoptotic ratio (Figures 1F,G) demonstrated a dose-dependent increase in osteoclast apoptosis, with profound apoptotic cell death observed at higher concentrations (50 and 100 μM) compared to the vehicle control. Finally, to elucidate the inhibitory effect of ECL on osteoclast function at the molecular level, we evaluated the expression of osteoclast-specific genes. The mRNA expression levels of pivotal osteoclastogenic markers, including Acp5, Calcr, Ctsk, and Nfatc1, were continuously downregulated following ECL treatment, with the maximum suppression achieved at the concentration of 50 μM, while a slight rebound was noted at 100 μM (Figure 1H). Taken together, these comprehensive *in vitro* functional and molecular evaluations demonstrate that ECL exerts a dual regulatory effect by simultaneously promoting osteoblastogenesis and suppressing osteoclastogenesis, and successfully identify 50 μM as the optimal pharmacological concentration for subsequent experiments. The modest decline or plateau observed at 100 μM across several assays may reflect early cytotoxic stress or off-target effects at suprapharmacological concentrations; we therefore restricted all subsequent mechanistic experiments to the sub-cytotoxic optima of 50 μM (ECL).

### Roles of ECLT in osteoblasts and osteoclasts *in vitro*

To comprehensively evaluate the pharmacological effects of the aglycone derivative Esculetin (ECLT) on bone remodeling *in vitro*, we similarly investigated its impact on osteogenic differentiation across a concentration gradient (0, 1, 5, 10, 50, and 100 μM). Having established the therapeutic potential of ECL, we next sought to characterize the osteogenic and anti-osteoclastogenic properties of its aglycone derivative, Esculetin (ECLT). Transcriptional profiling of primary osteoblasts revealed that ECLT treatment markedly elevated the mRNA levels of critical osteogenic markers, including Alp, Col1a, Ocn, and Runx2 (Figure 2A). This upregulation progressed in a dose-dependent fashion, with expression peaking sharply at the 10 μM dose. Consistent with these genetic alterations, macroscopic evaluation of Alizarin Red S (ARS) staining confirmed that ECLT profoundly enhanced calcium deposition and extracellular matrix mineralization, again demonstrating maximum functional efficacy at 10 μM (Figure 2B). We further explored the influence of ECLT on osteoblast expansion and survival. Flow cytometric assessment of EdU incorporation (Figure 2C) and its corresponding statistical quantification (Figure 2D) indicated a robust enhancement in cell proliferation, which reached its zenith at 10 μM but notably diminished at higher concentrations (50 and 100 μM). Similarly, cellular viability assays confirmed the biocompatibility of ECLT, showing a synchronized peak in overall osteoblast viability at 10 μM without evident cytotoxicity across the tested spectrum (Figure 2E). Biologically, the sharper optimum of ECLT at 10 μM is consistent with its aglycone structure, which likely achieves maximal osteogenic activity at a lower concentration than its glycoside parent, whereas higher concentrations may begin to engage stress responses.

**FIGURE 2:**
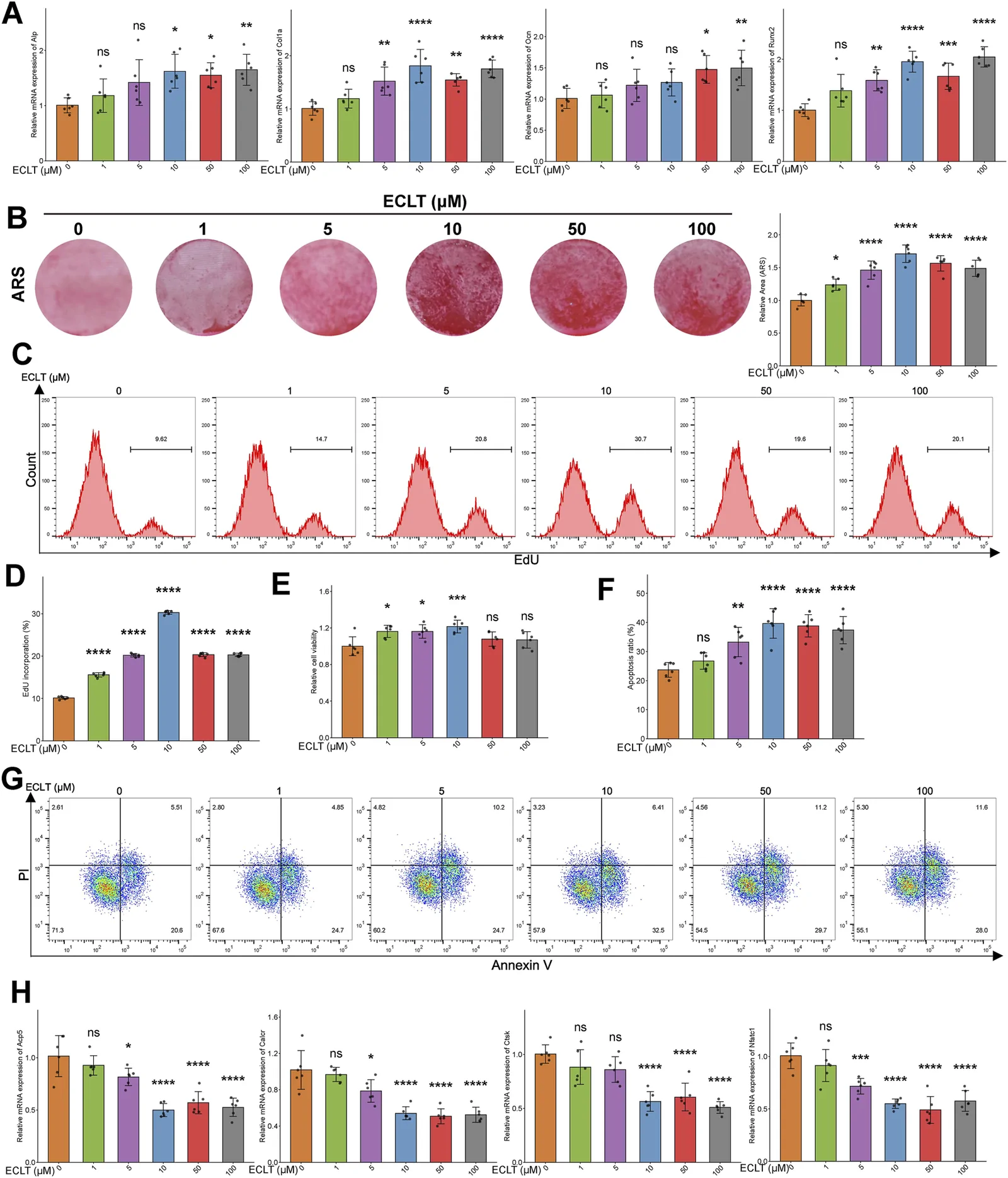
Esculetin (ECLT) dose-dependently promotes osteoblastogenesis and inhibits osteoclastogenesis *in vitro*. **(A)** Relative mRNA expression of osteogenic genes (Alp, Col1a, Ocn, Runx2) in osteoblasts following ECLT treatment. **(B)** Representative ARS staining images and corresponding quantification of calcium nodules. **(C,D)** EdU incorporation assay depicting osteoblast proliferation. **(E)** Cell viability of osteoblasts treated with ECLT. **(F,G)** Quantitative analysis **(F)** and representative Annexin V/PI flow cytometry plots **(G)** of osteoclast apoptosis. **(H)** Relative mRNA expression of osteoclastogenic genes (Acp5, Calcr, Ctsk, Nfatc1). n = 6 per group. Each dot represents an independent biological replicate (well), which represents the average value of three technical replicates, and data are presented as mean ± SD (error bars). Differences among groups were assessed by one-way ANOVA followed by Tukey’s *post hoc* test; asterisks denote comparisons with the vehicle control (0 μM): *P < 0.05, **P < 0.01, ***P < 0.001, ****P < 0.0001; ns, not significant. Exact adjusted P values are provided in Supplementary Table S2.

To determine whether ECLT shares the anti-resorptive characteristics of its parent compound, we evaluated its direct impact on RAW264.7-derived osteoclasts. The induction of osteoclast apoptosis was monitored via Annexin V/PI double staining. Both the quantitative statistical analysis (Figure 2F) and the representative flow cytometry dot plots (Figure 2G) depicted a significant escalation in the proportion of apoptotic cells. This pro-apoptotic response was highly sensitive to ECLT, reaching a maximum induction at 10 μM that was maintained at higher doses. At the transcriptional level, ECLT administration effectively crippled osteoclastogenesis by suppressing the expression of lineage-specific markers, such as Acp5, Calcr, Ctsk, and Nfatc1. The most substantial and stable transcriptional inhibition was consistently observed at the 10 μM concentration (Figure 2H). Collectively, these *in vitro* data underline the potent capacity of ECLT to rebalance bone metabolism by simultaneously stimulating osteoblast function and curtailing osteoclast survival. Consequently, 10 μM was established as the optimal pharmacological concentration for ECLT in all subsequent targeted comparative experiments. The parallel induction of apoptosis and suppression of osteoclastogenic transcription suggest that ECLT restrains the resorptive arm of remodeling at both the survival and the differentiation levels of the osteoclast lineage.

### Co-administration of ECL and ECLT yields enhanced therapeutic efficacy *in vitro*

Building upon the identification of optimal single-agent dosages (50 μM for ECL and 10 μM for ECLT), we subsequently investigated whether combining these two natural coumarin derivatives could elicit an additive or enhanced therapeutic effect. The *in vitro* cellular models were categorized into four comparative groups: vehicle control (PBS), ECL monotherapy, ECLT monotherapy, and the combination treatment (ECL + ECLT). Initial assessments of general cellular health revealed that the co-administration regimen was associated with the highest osteoblast viability among the groups, although the difference from each monotherapy did not reach statistical significance (Figure 3A). When evaluating the process of osteogenic differentiation, transcriptional profiling demonstrated that the ECL + ECLT combination robustly upregulated crucial bone-forming genes (Figure 3B). However, it is important to note that while Alp and Ocn expressions were significantly higher than those in either monotherapy, the further increases in Col1a and Runx2 represented numerical enhancements that did not reach statistical significance compared to the individual treatments. This amplified genetic activation translated directly into superior functional mineralization; macroscopic and quantitative analyses of Alizarin Red S (ARS) staining confirmed that the concurrent treatment elicited extensive calcium nodule deposition (Figure 3C). Although the combination was statistically superior to ECLT, the numerical increase in mineralization over ECL monotherapy did not reach statistical significance. To further dissect the cellular dynamics driving this expanded osteoblast pool, we tracked DNA synthesis utilizing the EdU incorporation assay. The flow cytometric profiles distinctly illustrated that the dual application of ECL and ECLT propelled osteoblast proliferation to its highest rate, substantially surpassing the proliferative capacities achieved by single-agent interventions (Figure 3D). Together, these data indicate that the two coumarins engage complementary aspects of osteoblast activation so that their combination produces a more complete anabolic response than either agent alone.

**FIGURE 3:**
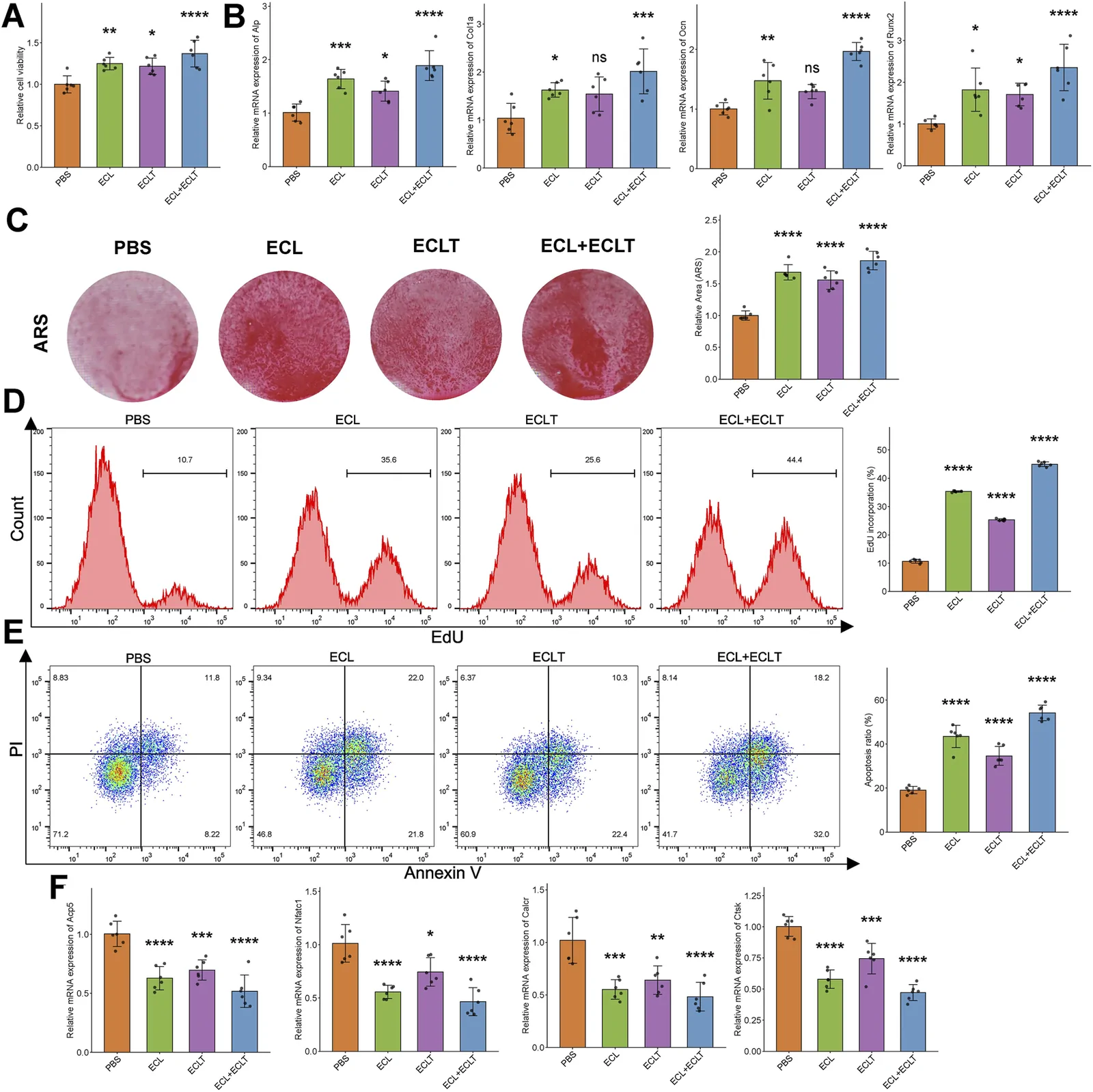
Co-administration of ECL and ECLT exerts enhanced therapeutic efficacy *in vitro*. **(A)** Relative cell viability of osteoblasts across different treatment groups (PBS, ECL, ECLT, and ECL + ECLT). **(B)** Upregulation of osteogenic marker genes (Alp, Col1a, Ocn, Runx2). **(C)** Representative ARS staining images and quantitative area analysis. **(D)** EdU incorporation assay and quantification showing enhanced osteoblast proliferation. **(E)** Annexin V/PI apoptosis assay demonstrating exacerbated osteoclast cell death in the combination group. **(F)** Relative mRNA expression of osteoclastogenic genes (Acp5, Nfatc1, Calcr, Ctsk). n = 6 per group. Each dot represents an independent biological replicate (well), which represents the average value of three technical replicates, and data are presented as mean ± SD (error bars). Differences among groups were assessed by one-way ANOVA followed by Tukey’s *post hoc* test; asterisks denote comparisons with the PBS control: *P < 0.05, **P < 0.01, ***P < 0.001, ****P < 0.0001; ns, not significant. Exact adjusted P values are provided in Supplementary Table S2.

Transitioning to the catabolic arm of bone remodeling, we evaluated the combinatorial capacity of these compounds to restrict RAW264.7-derived osteoclasts. Flow cytometric quantification via Annexin V/PI double staining indicated that the ECL + ECLT combination severely exacerbated osteoclast cell death, triggering a more profound and widespread apoptotic response than either ECL or ECLT in isolation (Figure 3E). In perfect alignment with these survival deficits, the essential transcriptional machinery of osteoclasts was most potently impaired by the dual treatment. Collectively, these comparative *in vitro* results illustrate that the combined application of ECL and ECLT strengthens the bidirectional regulation of bone homeostasis, providing a therapeutic profile that was greater than either monotherapy in several key functional readouts and laying a foundation for subsequent *in vivo* validation.

### Co-administration of ECL and ECLT markedly ameliorates systemic inflammation, oxidative stress, and bone turnover imbalances *in vivo*

To evaluate the translational potential of our *in vitro* findings, we proceeded to investigate the systemic therapeutic efficacy of ECL and ECLT utilizing two distinct *in vivo* murine models: ovariectomy (OVX)-induced postmenopausal osteoporosis and denervation (DEN)-induced disuse osteoporosis. We first assessed the systemic inflammatory and oxidative stress milieu in the OVX cohort. Serum ELISA analyses revealed that estrogen deprivation triggered a profound systemic inflammatory response, evidenced by drastically elevated circulating levels of pro-inflammatory cytokines (IL-6, IL-1β, and TNF-α) and a concurrent reduction in the anti-inflammatory cytokine IL-10 (Figure 4A). Concomitantly, OVX mice exhibited severe systemic oxidative stress, characterized by increased malondialdehyde (MDA) accumulation and depleted superoxide dismutase (SOD) activity (Figure 4B). While monotherapy with either ECL or ECLT significantly mitigated these pathological shifts, the combinatorial regimen (OVX + ECL + ECLT) achieved a remarkably superior rescue effect. The dual administration effectively normalized the inflammatory and oxidative stress profiles, restoring them to levels closely approximating those of the healthy vehicle control group. Because pro-inflammatory cytokines both stimulate osteoclastogenesis and suppress osteoblast activity, attenuation of this systemic milieu provides a plausible upstream mechanism through which the compounds rebalance bone turnover.

**FIGURE 4:**
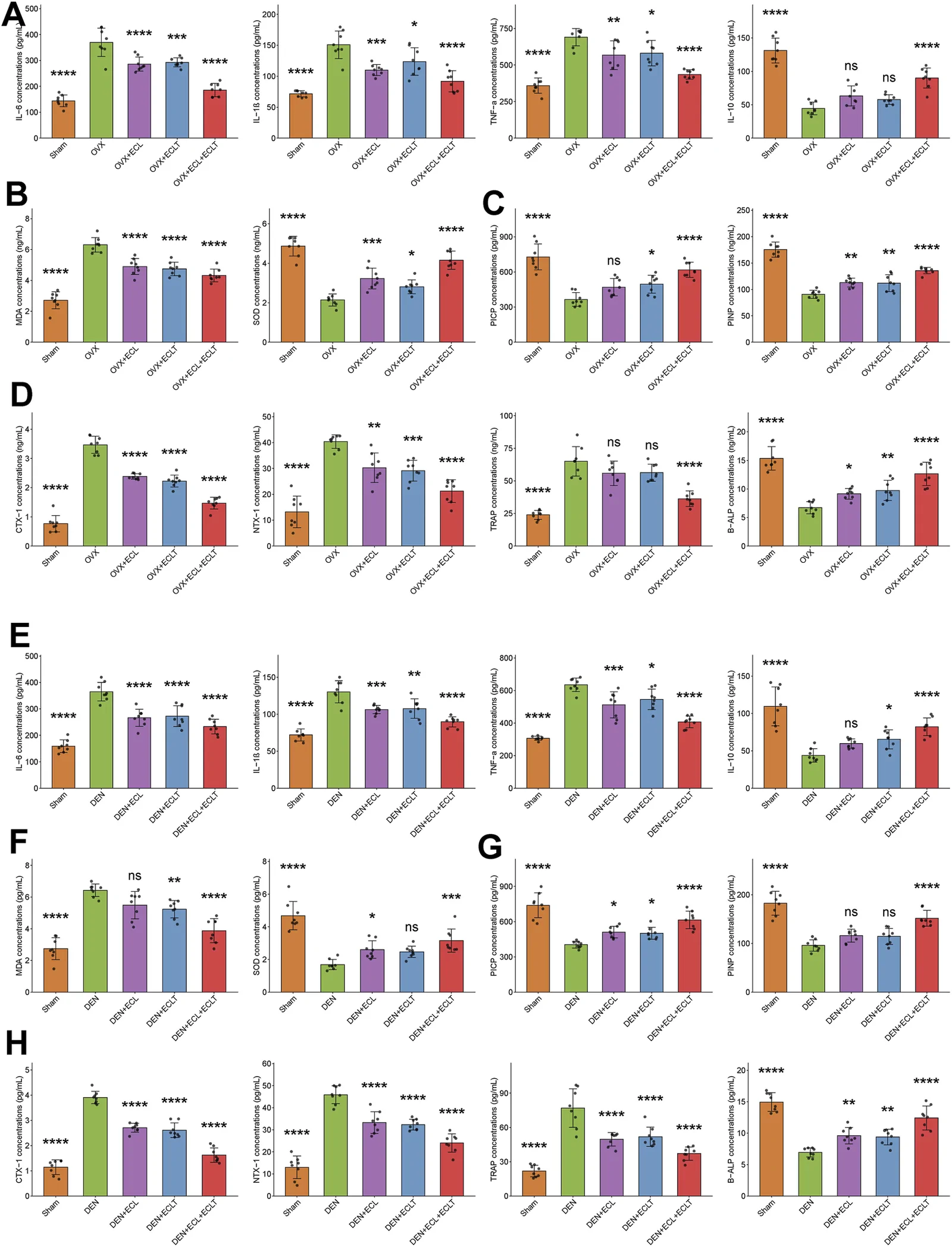
Combinatorial treatment of ECL and ECLT ameliorates systemic inflammation, oxidative stress, and bone turnover imbalances *in vivo*. **(A–D)** Serum ELISA biochemical analysis in the OVX model: **(A)** Inflammatory cytokines (IL-6, IL-1β, TNF-α, IL-10); **(B)** Oxidative stress markers (MDA, SOD); **(C)** Bone formation markers (PICP, PINP); **(D)** Bone resorption markers (CTX-1, NTX-1, TRAP) and bone alkaline phosphatase (B-ALP). **(E–H)** Serum ELISA biochemical analysis in the DEN model: **(E)** Inflammatory cytokines; **(F)** Oxidative stress markers; **(G)** Bone formation markers; **(H)** Bone resorption markers and B-ALP. n = 8 per group. Each dot represents one replicate animal, and data are presented as mean ± SD (error bars). Differences among groups were assessed by one-way ANOVA followed by Tukey’s *post hoc* test; asterisks denote comparisons with the OVX **(A–D)** or DEN **(E–H)** group: *P < 0.05, **P < 0.01, ***P < 0.001, ****P < 0.0001; ns, not significant. Exact adjusted P values are provided in Supplementary Table S2.

We subsequently quantified serum biochemical markers to directly monitor systemic bone turnover dynamics. Estrogen deficiency precipitated a marked uncoupling of bone remodeling, severely depressing bone formation while drastically accelerating bone resorption. Specifically, the circulating levels of crucial bone formation indices, including procollagen I C-terminal propeptide (PICP), procollagen I N-terminal propeptide (PINP) (Figure 4C), and bone alkaline phosphatase (B-ALP) (Figure 4D), were profoundly diminished in the OVX group. Conversely, systemic markers of osteoclast-mediated bone resorption, namely, C-terminal and N-terminal telopeptides of type I collagen (CTX-1 and NTX-1) alongside tartrate-resistant acid phosphatase (TRAP), were dramatically elevated (Figure 4D). Consistent with our *in vitro* observations, both ECL and ECLT treatments effectively reversed this catabolic imbalance. Most notably, the combined treatment was most effective, restoring bone formation markers and suppressing resorptive indicators to the greatest extent among all therapeutic interventions. The concordant movement of formation and resorption markers toward sham levels indicates a genuine shift in the remodeling balance rather than a selective suppression of a single arm.

To rigorously validate whether this combinatorial regimen confers broad-spectrum osteoprotection independent of the initial pathological trigger, we parallelly examined the DEN model of disuse osteoporosis. Mechanical unloading induced by sciatic nerve transection resulted in a systemic catabolic cascade strikingly analogous to the OVX model. Specifically, DEN mice displayed intense systemic inflammation and oxidative damage (Figures 4E,F), coupled with a profound deterioration in bone turnover homeostasis, as indicated by suppressed bone formation (PICP, PINP, B-ALP) and surging bone resorption (CTX-1, NTX-1, TRAP) (Figures 4G,H). Strikingly mirroring the outcomes observed in the estrogen-deprived cohort, the administration of ECL and ECLT effectively blunted these adverse systemic alterations. Once again, the combinatorial application (DEN + ECL + ECLT) elicited the most pronounced combined benefits, largely re-establishing systemic metabolic equilibrium, reducing inflammation, alleviating oxidative stress, and rebalancing the bone remodeling cascade across distinct etiologies of bone loss. The convergence of these systemic effects across a hormonal and a mechanical model suggests that the compounds act on shared inflammatory and oxidative pathways rather than on a single endocrine axis.

### Combinatorial therapy of ECL and ECLT prevents histological bone deterioration and re-establishes cellular remodeling equilibrium *in vivo*

To directly assess the protective effects of ECL and ECLT on bone microarchitecture at the tissue level, comprehensive histological evaluations were performed on the distal femurs of both the OVX (Figures 5A,B) and DEN cohorts (Figures 6A,B). Masson’s trichrome and Toluidine blue staining highlighted a severe depletion of the collagenous matrix and proteoglycan ground substance in these disease models. While monotherapy with either ECL or ECLT visibly mitigated these structural deteriorations, preserving trabecular connectivity and matrix density to a moderate extent, the combinatorial treatment (ECL + ECLT) delivered a profoundly superior structural rescue. In both osteoporotic models, the dual therapy maintained a dense, well-connected trabecular architecture and a rich collagen network that closely resembled the healthy Sham groups. The preservation of both collagenous matrix and the osteoid–osseous transition indicates that treatment protected the bone-forming surface in addition to limiting resorptive erosion.

**FIGURE 5:**
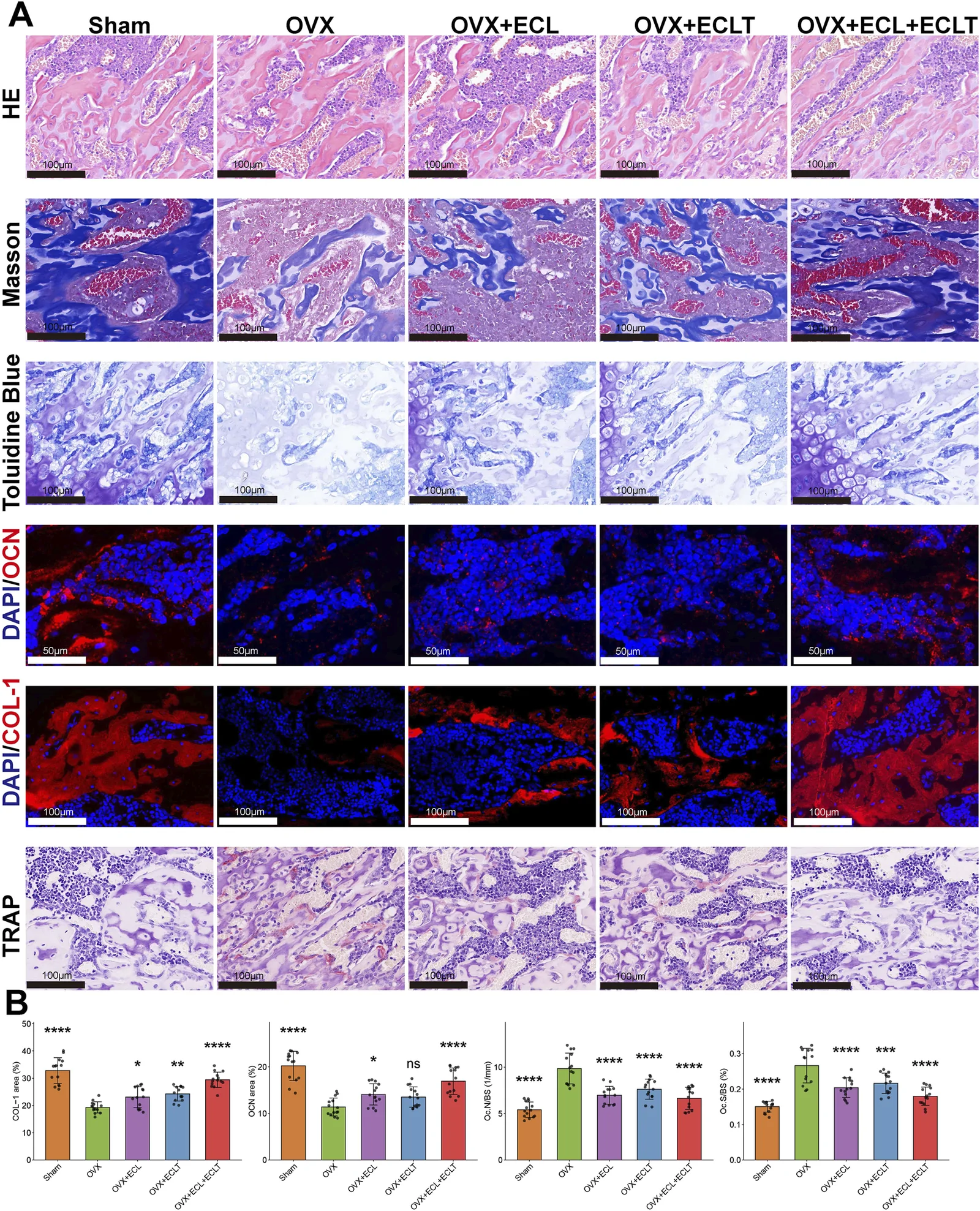
Combinatorial therapy of ECL and ECLT prevents histological bone deterioration and re-establishes cellular remodeling equilibrium in the OVX model. **(A)** Representative histological and immunofluorescence images of distal femurs from Sham, OVX, OVX + ECL, OVX + ECLT, and OVX + ECL + ECLT groups. From top to bottom: Hematoxylin and Eosin (H&E), Masson’s trichrome, Toluidine Blue, immunofluorescence for OCN (red) and COL-1 (red) with DAPI counterstain (blue), and TRAP staining. **(B)** Quantitative histomorphometric analysis of COL-1 area (%), OCN area (%), osteoclast number per bone surface (Oc.N/BS), and osteoclast surface per bone surface (Oc.S/BS). n = 14 per group. Each dot represents one replicate animal, and data are presented as mean ± SD (error bars). Differences among groups were assessed by one-way ANOVA followed by Tukey’s *post hoc* test; asterisks denote comparisons with the OVX group: *P < 0.05, **P < 0.01, ***P < 0.001, ****P < 0.0001; ns, not significant. Exact adjusted P values are provided in Supplementary Table S2.

**FIGURE 6:**
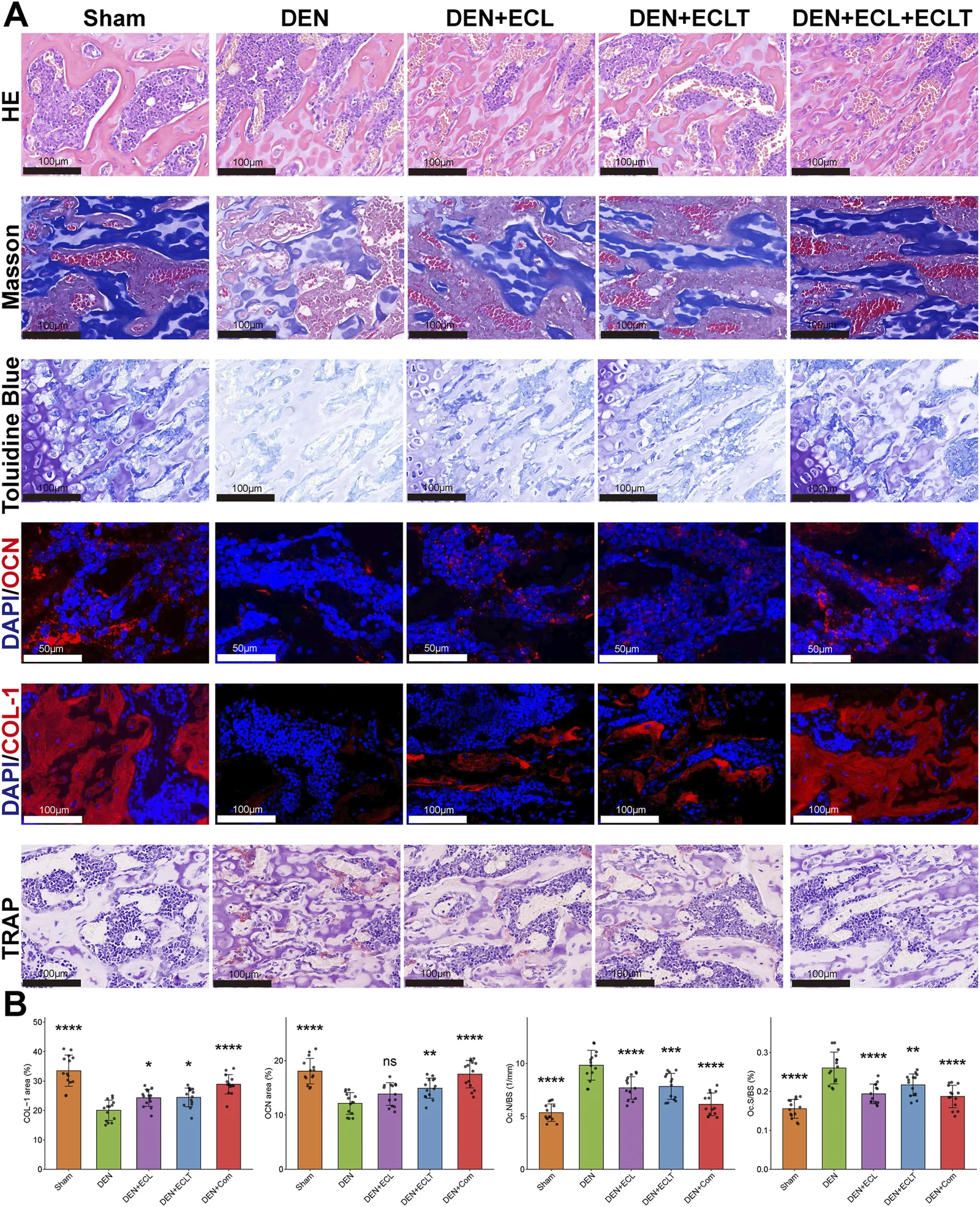
Combinatorial therapy of ECL and ECLT prevents histological bone deterioration and re-establishes cellular remodeling equilibrium in the DEN model. **(A)** Representative histological and immunofluorescence images of distal femurs from Sham, DEN, DEN + ECL, DEN + ECLT, and DEN + ECL + ECLT groups. From top to bottom: Hematoxylin and Eosin (H&E), Masson’s trichrome, Toluidine Blue, immunofluorescence for OCN (red) and COL-1 (red) with DAPI counterstain (blue), and TRAP staining. **(B)** Quantitative histomorphometric analysis of COL-1 area (%), OCN area (%), osteoclast number per bone surface (Oc.N/BS), and osteoclast surface per bone surface (Oc.S/BS). n = 14 per group. Each dot represents one replicate animal, and data are presented as mean ± SD (error bars). Differences among groups were assessed by one-way ANOVA followed by Tukey’s *post hoc* test; asterisks denote comparisons with the DEN group: *P < 0.05, **P < 0.01, ***P < 0.001, ****P < 0.0001; ns, not significant. Exact adjusted P values are provided in Supplementary Table S2.

We next utilized immunofluorescence to quantitatively evaluate the *in vivo* synthetic function of osteoblasts. The structural protein Collagen Type I (COL-1) and the mature osteoblast marker Osteocalcin (OCN) were prominently expressed along the trabecular surfaces in Sham mice, but their fluorescent intensities were drastically diminished following ovariectomy or denervation. Treatment with either ECL or ECLT alone elicited a significant, albeit partial, recovery in the expression of these osteogenic markers. In striking contrast, the co-administration of ECL and ECLT most effectively enhanced osteoblast-mediated matrix synthesis. Quantitative histomorphometric analysis of the immunofluorescence area confirmed that the combination therapy most effectively restored the expression levels of COL-1 and OCN, driving them remarkably close to baseline physiological levels across both etiologies of bone loss (Figures 5A,B, 6A,B). Because OCN marks the mature, mineralization-competent osteoblast whereas COL-1 reflects matrix output, their coordinated restoration implies recovery of osteoblast function rather than a nonspecific increase in cell number.

Complementing the osteogenic findings, tartrate-resistant acid phosphatase (TRAP) staining was employed to visualize and quantify *in vivo* osteoclast activity. Consistent with the circulating bone turnover markers, histological sections from the OVX and DEN vehicle-treated groups exhibited massive infiltration of hyperactive, multinucleated TRAP-positive osteoclasts lining the eroded trabecular surfaces (Figures 5A, 6A). Monotherapy with ECL or ECLT significantly constrained this cellular catabolic drive. However, the most pronounced anti-resorptive effect was observed with the combination treatment. Collectively, these histological and cellular *in vivo* data provide compelling evidence that the combined application of ECL and ECLT helps preserve bone mass by concurrently promoting osteogenesis and restraining osteoclast formation. The reduction in both osteoclast number (Oc.N/BS) and osteoclast surface (Oc.S/BS) further indicates that the combination limits both the generation and the bone-surface occupancy of the resorptive compartment.

### Combined intervention of ECL and ECLT preserves 3D microstructure and improves biomechanical bone strength

To rigorously quantify the protective effects of the therapeutic interventions on macroscopic skeletal geometry, high-resolution micro-computed tomography (Micro-CT) and biomechanical analysis were performed on the distal femurs (Figures 7A,B, 8A,B). Three-dimensional reconstructive images visually demonstrated that both OVX (Figure 7A) and DEN (Figure 8A) precipitated a catastrophic deterioration of the trabecular network, characterized by a porous, disjointed, and thinned structural appearance. Quantitative analysis corroborated these macroscopic observations: vehicle-treated OVX and DEN mice exhibited severe declines in bone volume fraction (BV/TV), trabecular number (Tb.N), trabecular thickness (Tb.Th), connectivity density (Conn.D), and cortical bone thickness (Cb.Th), accompanied by a drastic pathological expansion in trabecular separation (Tb.Sp). Although monotherapy with either ECL or ECLT provided moderate skeletal protection, partially rescuing several microstructural parameters, the combinatorial treatment (ECL + ECLT) delivered the greatest restorative effect. In both the estrogen-deficient and mechanically unloaded microenvironments, the dual therapy robustly preserved the three-dimensional trabecular and cortical bone mass, returning most critical microarchitectural parameters toward Sham levels; in the DEN model, several parameters remained significantly lower than Sham (Figures 7A, 8A). Preservation of connectivity density and trabecular number is particularly relevant to fracture resistance, because these parameters govern load transfer through the trabecular network.

**FIGURE 7:**
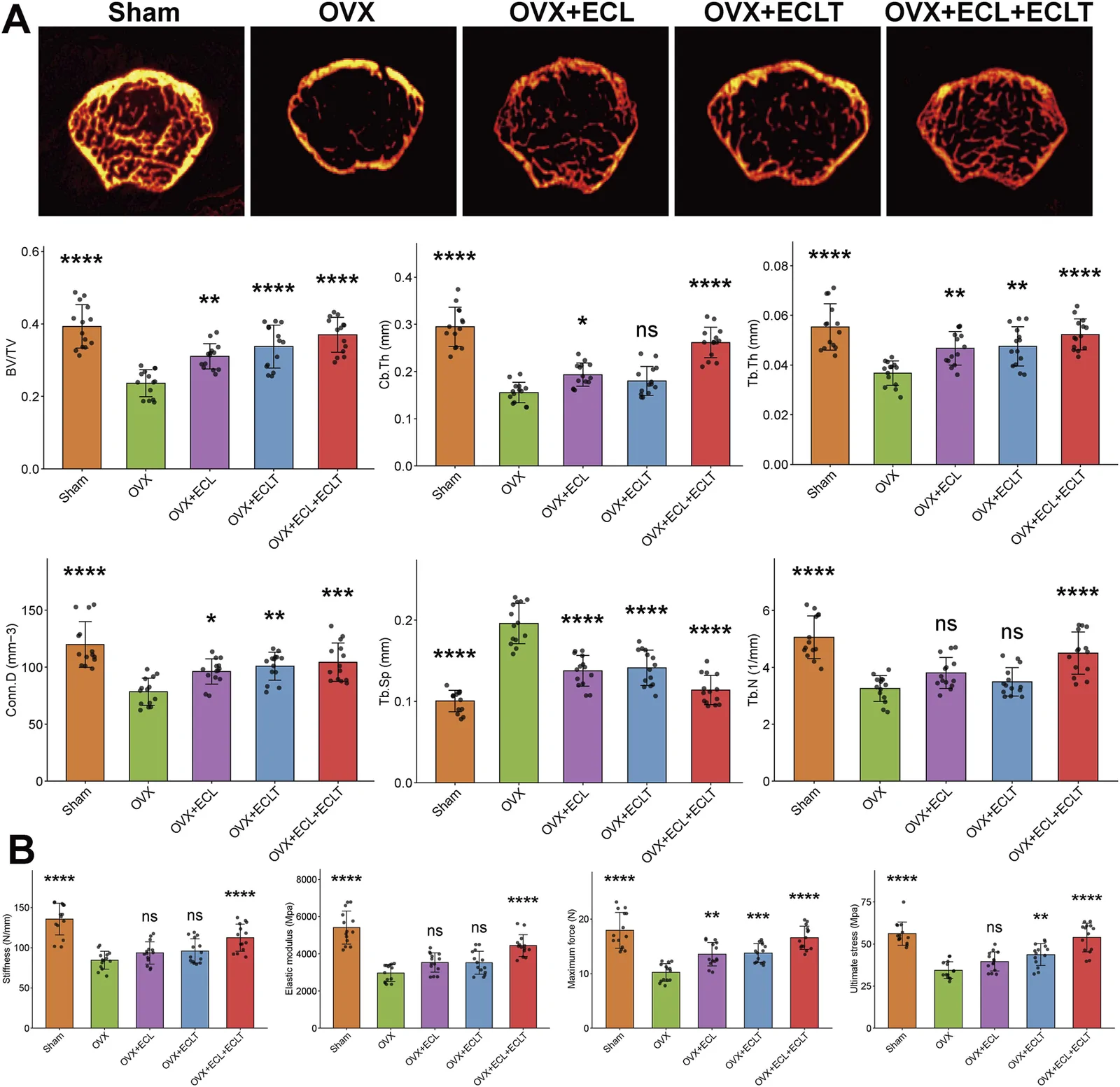
Combined intervention with ECL and ECLT preserves 3D microstructural integrity and improves biomechanical strength in OVX mice. **(A)** Representative 3D Micro-CT reconstructions of distal femur trabecular bone, alongside quantitative analysis of critical microarchitectural parameters: bone volume fraction (BV/TV), cortical bone thickness (Cb.Th), trabecular thickness (Tb.Th), connectivity density (Conn.D), trabecular separation (Tb.Sp), and trabecular number (Tb.N). **(B)** Biomechanical properties determined by three-point bending tests, including stiffness, elastic modulus, maximum force, and ultimate stress. n = 14 per group. Each dot represents one replicate animal, and data are presented as mean ± SD (error bars). Differences among groups were assessed by one-way ANOVA followed by Tukey’s *post hoc* test; asterisks denote comparisons with the OVX group: *P < 0.05, **P < 0.01, ***P < 0.001, ****P < 0.0001; ns, not significant. Exact adjusted P values are provided in Supplementary Table S2.

**FIGURE 8:**
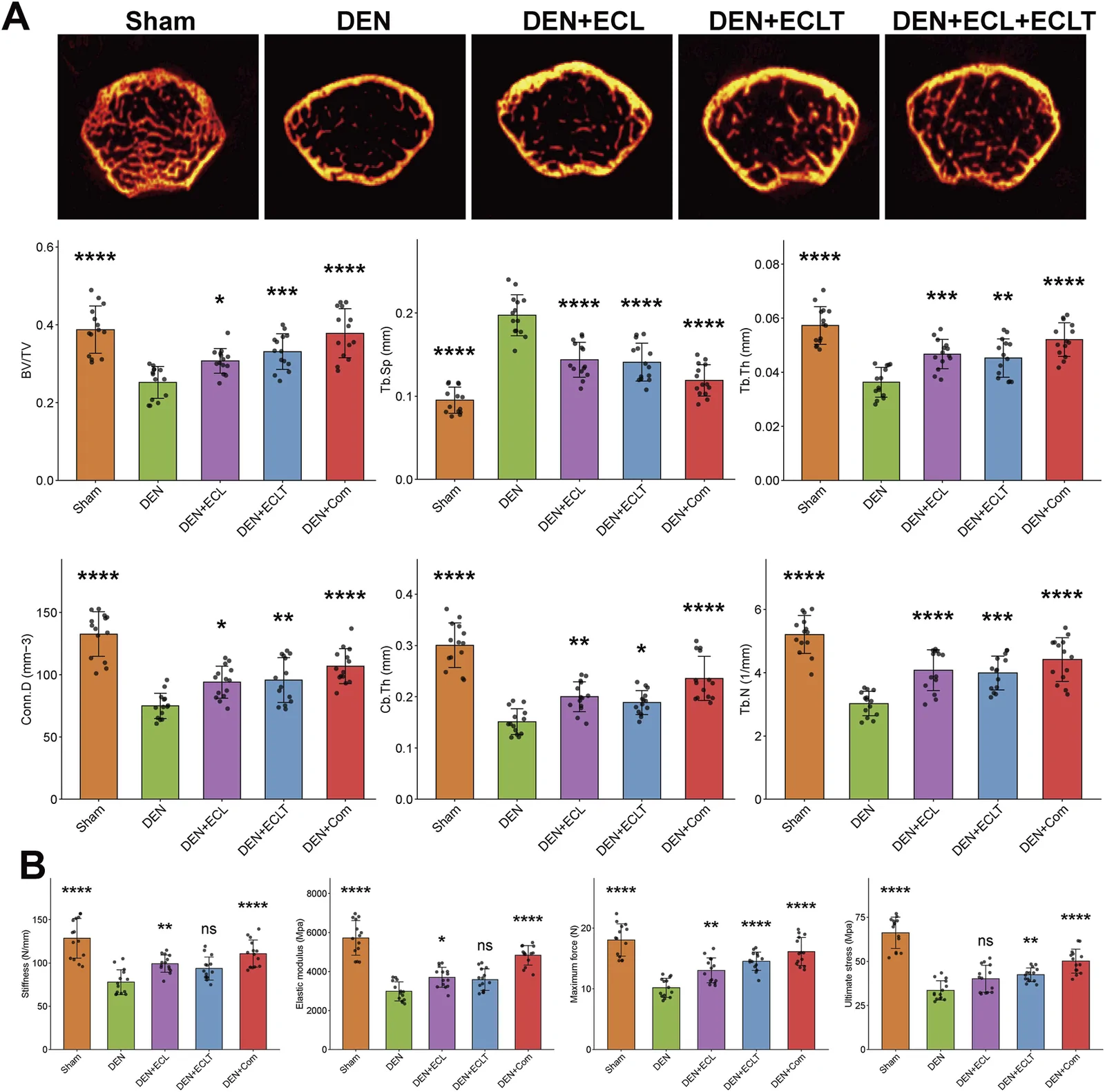
Combined intervention with ECL and ECLT preserves 3D microstructural integrity and improves biomechanical strength in DEN mice. **(A)** Representative 3D Micro-CT reconstructions of distal femur trabecular bone, alongside quantitative analysis of critical microarchitectural parameters: bone volume fraction (BV/TV), cortical bone thickness (Cb.Th), trabecular thickness (Tb.Th), connectivity density (Conn.D), trabecular separation (Tb.Sp), and trabecular number (Tb.N). **(B)** Biomechanical properties determined by three-point bending tests, including stiffness, elastic modulus, maximum force, and ultimate stress. n = 14 per group. Each dot represents one replicate animal, and data are presented as mean ± SD (error bars). Differences among groups were assessed by one-way ANOVA followed by Tukey’s *post hoc* test; asterisks denote comparisons with the DEN group: *P < 0.05, **P < 0.01, ***P < 0.001, ****P < 0.0001; ns, not significant. Exact adjusted P values are provided in Supplementary Table S2.

To determine whether this profound 3D structural preservation translated into functional skeletal benefits, we subjected the femurs to a three-point bending biomechanical test. As anticipated, the profound microarchitectural decay in the OVX and DEN cohorts culminated in severe functional deficits, rendering the bones highly fragile and susceptible to fracture. This was evidenced by drastic reductions in mechanical parameters, including maximum force, stiffness, elastic modulus, and ultimate stress (Figures 7B, 8B). While administration of ECL or ECLT alone conferred varying degrees of biomechanical improvement, with certain parameters remaining statistically insignificant compared to the disease models, the concurrent application of ECL and ECLT most consistently rescued the mechanical integrity of the femurs. The combinatorial regimen substantially improved the load-bearing capacity, elasticity, and overall structural durability of the bones across both osteoporotic etiologies (Figures 7B, 8B). Taken together, these comprehensive radiological and biomechanical data compellingly demonstrate that the ECL and ECLT combination therapy not only prevents bone mass depletion but also meticulously safeguards the functional and mechanical quality of the skeleton, highlighting its promising translational potential for osteoporosis management.

## Discussion

Osteoporosis is a prevalent systemic skeletal disorder characterized by the progressive deterioration of bone microarchitecture and uncoupled bone remodeling. Current pharmacological interventions predominantly target either bone resorption or formation, which can lead to secondary suppression of the opposing process and long-term adverse effects (Wang M. et al., 2026; Xia et al., 2026). In the present study, we systematically investigated the pharmacological effects of Esculin (ECL) and its aglycone derivative Esculetin (ECLT), naturally occurring coumarins derived from Cortex Fraxini (Liao et al., 2026; Xin et al., 2026). We demonstrated that both ECL and ECLT exert bidirectional protective actions by simultaneously promoting osteoblastogenesis and suppressing osteoclastogenesis *in vitro*. Furthermore, their co-administration ameliorated bone loss and substantially improved biomechanical strength across distinct *in vivo* models of postmenopausal and disuse osteoporosis.

The delicate equilibrium of bone homeostasis relies on the tightly coupled functions of bone-forming osteoblasts and bone-resorbing osteoclasts. Our cellular assays revealed that ECL (50 μM) and ECLT (10 μM) independently stimulated osteoblast proliferation and extracellular matrix mineralization without inducing cytotoxicity. This anabolic effect was driven by transcriptional upregulation of crucial osteogenic markers. Concurrently, both compounds impaired osteoclast formation, as evidenced by induced osteoclast apoptosis, reduced TRAP-positive multinucleated cell area, and downregulation of key resorption-related genes. These findings align closely with previous evidence that esculetin promotes new bone formation and inhibits osteoclastogenesis in ovariectomized rats (Baek et al., 2015; Liu et al., 2018). Additionally, metabolomics analysis has identified esculin as a key metabolite contributing to the amelioration of glucocorticoid-induced bone loss (Yin et al., 2023). Previous investigations have predominantly focused on the pharmacological actions of individual compounds. Herein, by employing a combinatorial strategy involving the concurrent administration of ECL and ECLT, our study extends this earlier work and establishes a preclinical rationale for their application as dual-action modulators of bone remodeling.

Systemic inflammation and chronic oxidative stress are fundamental pathological triggers that uncouple bone remodeling and accelerate skeletal decay (Liu et al., 2022; An et al., 2026; Kou et al., 2026; Wang Y. et al., 2026). In both the ovariectomy (OVX) and denervation (DEN) models, mice exhibited profound inflammatory surges, characterized by elevated pro-inflammatory cytokines (IL-6, IL-1β, TNF-α) and diminished anti-inflammatory IL-10. This was accompanied by severe oxidative damage, indicated by increased malondialdehyde (MDA) accumulation and depleted superoxide dismutase activity. Treatment with ECL and ECLT potently reversed these systemic disruptions, restoring inflammatory and oxidative stress profiles to near-physiological levels. Previous pharmacological profiling has highlighted that esculin and esculetin possess tremendous antioxidative and anti-inflammatory capacities, primarily operating through the activation of the Nrf2/HO-1 antioxidant pathway and the inhibition of the NF-κB and MAPK inflammatory signaling cascades (Jung et al., 2022; Cai and Cai, 2023; Ju et al., 2024; Luo et al., 2024; Kou et al., 2026). The mitigation of these systemic stressors in our models seamlessly translated into a rebalanced bone turnover, evidenced by the normalization of circulating bone formation markers and the suppression of resorptive indicators.

A key observation of this study is the enhanced efficacy achieved through the combined administration of ECL and ECLT. Natural coumarin derivatives frequently face translational challenges due to poor aqueous solubility, rapid phase II metabolism, and characteristically low oral bioavailability (Kim et al., 2026; Liao et al., 2026). However, combinatorial strategies leveraging these compounds have been successfully shown to enhance therapeutic binding affinities and block dual intracellular signaling pathways in other pathological contexts, such as colorectal cancer (Liao et al., 2026). It should also be noted that esculin can be hydrolyzed to esculetin by the gut microbiota and intestinal enzymes, so concurrent oral administration may in principle alter the systemic exposure to the aglycone; dedicated pharmacokinetic evaluation of the combination is therefore warranted and is acknowledged in our limitations. In our skeletal models, the co-administration regimen outperformed either monotherapy, resulting in greater osteoblast-mediated matrix synthesis (COL-1 and OCN expression) and more marked suppression of osteoclasts.

Importantly, this combined cellular rescue effectively translated into macroscopic skeletal protection across both hormonal (OVX) and mechanical unloading (DEN) etiologies of osteoporosis. The dual therapy largely prevented three-dimensional microarchitectural decay, maintaining trabecular connectivity and preserving cortical bone thickness. Consequently, the mechanical integrity of the femurs was substantially improved. This broad osteoprotection suggests that the combinatorial regimen mitigates the downstream, convergent mechanism of osteoclast overactivation shared by these models.

Despite these highly encouraging structural and functional outcomes, our study has certain limitations. While we confirmed the profound systemic anti-inflammatory and dual cellular regulatory effects, the precise molecular targets and intracellular signaling crosstalk underlying any interactive effect between ECL and ECLT remain to be fully elucidated. Future investigations should employ advanced transcriptomic profiling and rigorous pharmacokinetic assessments to decode their combined metabolic stability and precise molecular mechanisms. Moreover, our mechanistic evidence at the cellular level is largely transcriptional, and protein- or pathway-level validation will be required to confirm these mechanisms. We also acknowledge several additional limitations: no treatment-only (healthy animal) groups or systematic toxicity assessments were included; a resorption-pit assay to directly measure osteoclast resorptive function was not performed; and pharmacokinetics, bioavailability, long-term toxicity, fracture outcomes, and head-to-head comparisons with established osteoporosis therapies were not evaluated. Accordingly, the present findings should be interpreted as preclinical proof of concept rather than as evidence of clinical efficacy. Additionally, while our *in vitro* data demonstrate significant changes in apoptosis and transcriptional profiles, these represent cellular-level observations rather than definitive proof of a direct causal mechanism or direct functional inhibition of osteoclast resorptive activity.

In conclusion, our findings systematically define the therapeutic roles of ECL and ECLT in bone metabolism. By concurrently promoting osteoblast function and suppressing osteoclast formation, their co-administration alleviated systemic inflammation and oxidative stress, thereby preserving three-dimensional bone microarchitecture and improving biomechanical strength. The combined application of these natural coumarins represents a promising bidirectional therapeutic strategy for the management of osteoporosis.

## Data Availability

The original contributions presented in the study are included in the article/Supplementary Material, further inquiries can be directed to the corresponding authors.
